# Effect of a combined brief narrative exposure therapy with case management versus treatment as usual in primary care for patients with traumatic stress sequelae following intensive care medicine: study protocol for a multicenter randomized controlled trial (PICTURE)

**DOI:** 10.1186/s13063-018-2853-7

**Published:** 2018-09-10

**Authors:** Jochen Gensichen, Susanne Schultz, Christine Adrion, Konrad Schmidt, Maggie Schauer, Daniela Lindemann, Natalia Unruh, Robert P. Kosilek, Antonius Schneider, Martin Scherer, Antje Bergmann, Christoph Heintze, Stefanie Joos, Josef Briegel, Andre Scherag, Hans-Helmut König, Christian Brettschneider, Thomas G. Schulze, Ulrich Mansmann, Klaus Linde, Dagmar Lühmann, Karen Voigt, Sabine Gehrke-Beck, Roland Koch, Bernhard Zwissler, Gerhard Schneider, Herwig Gerlach, Stefan Kluge, Thea Koch, Andreas Walther, Oxana Atmann, Jan Oltrogge, Maik Sauer, Julia Schnurr, Thomas Elbert, on behalf of the PICTURE Study Group, Christine Adrion, Matthias Angstwurm, Oxana Atmann, Michael Bauer, Antje Bergmann, Laura Bertram, Gerhard Bielmeier, Ralph Bogdanski, Franz Brettner, Christian Brettschneider, Josef Briegel, Martin Bürkle, Ulrike Dorn, Thomas Elbert, Kristin Elbs, Peter Falkai, Richard Fisch, Hans Förstl, Benjamin Fohr, Martin Franz, Lorenz Frey, Patrick Friederich, Jürgen Gallinat, Sabine Gehrke-Beck, Jochen Gensichen, Herwig Gerlach, Andreas Güldner, Hanna Hardt, Christoph Heintze, Andreas Heinz, Axel Heller, Christian Heymann, Petra Hoppmann, Volker Huge, Michael Irlbeck, Ulrich Jaschinski, Dominik Jarczak, Stefanie Joos, Elisabeth Kaiser, Melanie Kerinn, Frank-Rainer Klefisch, Stefan Kluge, Tina Knäfel, Roland Koch, Thea Koch, Hans-Helmut König, Robert Kosilek, Martin Krüger, Peter Lackermeier, Karl-Ludwig Laugwitz, Yvonne Lemke, Achim Lies, Klaus Linde, Daniela Lindemann, Dagmar Lühmann, Ulrich Mansmann, Stephanie May, Thorsten Möller, Ludwig Ney, Jan Oltrogge, Wulf Pankow, Sergi Papiol, Maximilian Ragaller, Nikolaus Rank, Lorenz Reill, Ulf-Dietrich Reips, Reimer Riessen, Grit Ringeis, Maik Sauer, Maggie Schauer, Gustav Schelling, Jörg Schelling, André Scherag, Martin Scherer, Konrad Schmidt, Antonius Schneider, Gerhard Schneider, Jürgen Schneider, Ralph Schneider, Julia Schnurr, Susanne Schultz, Thomas G. Schulze, Karin Schumacher, Peter Spieth, Kerstin Theisen, Franka Thurm, Aline Ulbricht, Natalia Unruh, Thomas Vogl, Karen Voigt, Andreas Walther, Dietmar Wassilowsky, Steffen Weber-Carstens, Roland Weierstall, Marion Weis, Georg Weiss, Harald Well, Christian Zöllner, Bernhard Zwissler

**Affiliations:** 1 Institute of General Practice and Family Medicine, University Hospital, LMU Munich, Pettenkoferstr. 8a, 80336 Munich, Germany; 2 Institute for Medical Information Processing, Biometry, and Epidemiology (IBE), LMU Munich, Marchioninistr. 15, 81377 Munich, Germany; 3 Institute of General Practice of the Charité, Universitätsmedizin Berlin, Campus Charité Mitte, Charitéplatz 1, 10117 Berlin, Germany; 4 Clinical Psychology, University of Konstanz, 78457 Konstanz, Germany; 5 Institute of General Practice, Technical University of Munich, Klinikum rechts der Isar, Orleansstr. 47, 81667 Munich, Germany; 6 Department of General Practice / Primary Care, University Medical Center Hamburg-Eppendorf, Haus West 37, Martinistr. 52, 20246 Hamburg, Germany; 7 Department of General Practice/Clinic of General Medicine – Medical clinic III, University Hospital Carl Gustav Carus, Technische Universität Dresden, Fetscherstr. 74, 01307 Dresden, Germany; 8 Institute for General Practice and Interprofessional Health Care, University Clinic Tübingen, Osianderstr. 5, 72076 Tübingen, Germany; 9 Department of Anaesthesiology, University Hospital, LMU Munich, Marchioninistr. 15, 81377 Munich, Germany; 10 Institute of Medical Statistics, Computer and Data Sciences, Jena University Hospital, Bachstr. 18, 07743 Jena, Germany; 11 Department of Health Economics and Health Services Research, University Medical Center Hamburg-Eppendorf, Martinistr. 52, 20246 Hamburg, Germany; 12 Institute of Psychiatric Phenomics and Genomics, University Hospital, LMU Munich, Marchioninistr. 15, 81377 Munich, Germany; 13 Clinic for Anesthesiology, Technical University of Munich, Klinikum rechts der Isar, Orleansstr. 47, 81667 Munich, Germany; 14 Clinic for Anesthesiology, Operative Intensive Care and Pain Management, Vivantes Klinikum Neukölln, Rudower Str. 49, 12351 Berlin, Germany; 15 Center for Anesthesiology and Intensive Care Medicine, University Medical Center Hamburg-Eppendorf, Martinistr. 52, 20246 Hamburg, Germany; 16 Clinic of Anesthesiology and Intensive Care Medicine, University Hospital Carl Gustav Carus, Technische Universität Dresden, Fetscherstr. 74, 01307 Dresden, Germany; 17 Clinic for Anesthesiology and Operative Intensive Care, Klinikum Stuttgart – Katharinenhospital, Kriegsbergerstr. 60, 70174 Stuttgart, Germany; 18 Institute of General Practice and Family Medicine, Jena University Hospital, Bachstr. 18, 07743 Jena, Germany

**Keywords:** Stress disorders (MeSH), Intensive care (MeSH), Non-pharmacological (NON-Mesh), Primary health care (MeSH), Randomized controlled trial (MeSH)

## Abstract

**Background:**

Traumatic events like critical illness and intensive care are threats to life and bodily integrity and pose a risk factor for posttraumatic stress disorder (PTSD). PTSD affects the quality of life and morbidity and may increase health-care costs. Limited access to specialist care results in PTSD patients being treated in primary care settings. Narrative exposure therapy (NET) is based on the principles of cognitive behavioral therapy and has shown positive effects when delivered by health-care professionals other than psychologists.

The primary aims of the PICTURE trial (from “PTSD after ICU survival”) are to investigate the effectiveness and applicability of NET adapted for primary care with case management in adults diagnosed with PTSD after intensive care.

**Methods/design:**

This is an investigator-initiated, multi-center, primary care-based, randomized controlled two-arm parallel group, observer-blinded superiority trial conducted throughout Germany. In total, 340 adult patients with a total score of at least 20 points on the posttraumatic diagnostic scale (PDS-5) 3 months after receiving intensive care treatment will be equally randomized to two groups: NET combined with case management and improved treatment as usual (iTAU). All primary care physicians (PCPs) involved will be instructed in the diagnosis and treatment of PTSD according to current German guidelines. PCPs in the iTAU group will deliver usual care during three consultations. In the experimental group, PCPs will additionally be trained to deliver an adapted version of NET (three sessions) supported by phone-based case management by a medical assistant. At 6 and 12 months after randomization, structured blinded telephone interviews will assess patient-reported outcomes.

The primary composite endpoint is the absolute change from baseline at month 6 in PTSD symptom severity measured by the PDS-5 total score, which also incorporates the death of any study patients. Secondary outcomes cover the domains depression, anxiety, disability, health-related quality-of-life, and cost-effectiveness. The principal analysis is by intention to treat.

**Discussion:**

If the superiority of the experimental intervention over usual care can be demonstrated, the combination of brief NET and case management could be a treatment option to relieve PTSD-related symptoms and to improve primary care after intensive care.

**Trial registration:**

ClinicalTrials.gov, NCT03315390. Registered on 10 October 2017.

German Clinical Trials Register, DRKS00012589. Registered on 17 October 2017.

**Electronic supplementary material:**

The online version of this article (10.1186/s13063-018-2853-7) contains supplementary material, which is available to authorized users.

## Background

In Germany, more than two million people are treated in intensive care units (ICUs) every year, more than 350,000 of whom undergo mechanical ventilation. These patients may suffer long-term functional, psychological, or medical sequelae [1–3], but there are only a limited number of treatment options [4]. Posttraumatic stress disorder (PTSD) is a common sequela (25–44%) of critical illness and ICU treatment and has a substantial impact on health-related quality of life and health-care-related costs [5, 6]. Systematic screening and early interventions in primary care may improve outcomes [7, 8]. In Germany, a guideline for treating patients with PTSD in primary care recommends supportive symptomatic pharmacologic therapy on a primary care level and referral to a specialist for psychotherapy and other non-pharmacological interventions [9]. However, access to psychiatric and psychotherapeutic specialty services, e.g. trauma therapists, is limited and waiting times are usually 5 months or longer [10, 11]. During this time, a primary care physician (PCP) is the main health-care professional attending to the patient. An effective psychological therapy for ICU-related PTSD applicable to primary care is needed [12].

### Trial rationale

Currently, the underlying mechanism of PTSD is assumed to be a disturbance in the organization and processing of memories of traumatic events, resulting in a separation of sensory, cognitive, and affective representations from the contextual and episodic memory system [13]. Patients who suffer from traumatic stressful experiences cannot clearly structure these events in chronological order and are, thus, unable to place the anxiety and helplessness associated with these events appropriately in time and space. Consequently, alarm responses can become activated by even small, subtle prompts. The resulting change affects the homeostasis of all physiological systems. The goal of psychotherapeutic interventions for PTSD is to teach survivors which cues relate to traumatic experiences in the past so that they no longer trigger an alarm response in the present.

Narrative exposure therapy (NET) is a specific form of psychotherapy for PTSD based on cognitive behavioral therapy [13]. During this treatment, the patient develops a narrative of traumatic events, which is meant to consolidate fragmented memories by setting these events into their respective context of time, place, and situation. NET typically consists of a session of psychoeducation, followed by a session in which the patient creates a graphical representation of their biography using a lifeline. Then, there are several sessions in which the patient recounts the stressful situations to recover contextual details of the traumatic event. NET is effective even when limited to only three to four sessions and also when delivered by health-care professionals other than psychotherapists [14–16].

In this study, a psychological intervention combines a brief version of NET adapted to primary care [13] with the principles of the chronic care model for special case management (telephone monitoring by the medical assistant or MA) [17]. The latter is one of the core components of this model. It includes case management focused on proactive patient symptom monitoring, clinical decision support for the PCP, and training for PCPs in evidence-based care.

A randomized controlled two-arm study of sepsis survivors (SMOOTH trial) enrolled 291 adult patients between February 2011 and December 2014 [18]. Patients were recruited from nine ICUs across Germany after having survived sepsis and randomized to usual primary care or to a 12-month intervention, consisting of usual primary care plus additional PCP and patient training, case management provided by study nurses, and clinical decision support for PCPs by consulting physicians. Based on the SMOOTH trial, which examined whether a primary care–based intervention improved health-related quality of life in adult sepsis survivors, we designed the PICTURE trial, which aims to improve traumatic stress sequela for post ICU-patients in a primary care setting [18].

## Methods/design

### Aims and objectives

The primary aims of the PICTURE trial (from “PTSD after ICU survival”) are to investigate the effectiveness, safety, and applicability of a brief NET-oriented primary care intervention combined with systematic trauma monitoring in ICU survivors compared to improved treatment as usual (iTAU), and to assess the maintenance of a possible treatment effect (defined as an improvement in PTSD-related symptoms) and applicability assessed at 6 and 12 months after baseline.

### Trial design and setting

PICTURE is a multi-center, two-arm parallel-group, observer-blinded, randomized, active-controlled superiority trial. The trial will be conducted in primary care practices across Germany. Trial management will be delivered by academic primary care institutes at university hospitals around Munich, Berlin, Hamburg, Dresden, Tübingen, and other areas. The primary care setting is associated with long-lasting doctor–patient relationships and coordination of health services, in accordance with the definition of Starfield et al. [19].

**Fig. 1:**
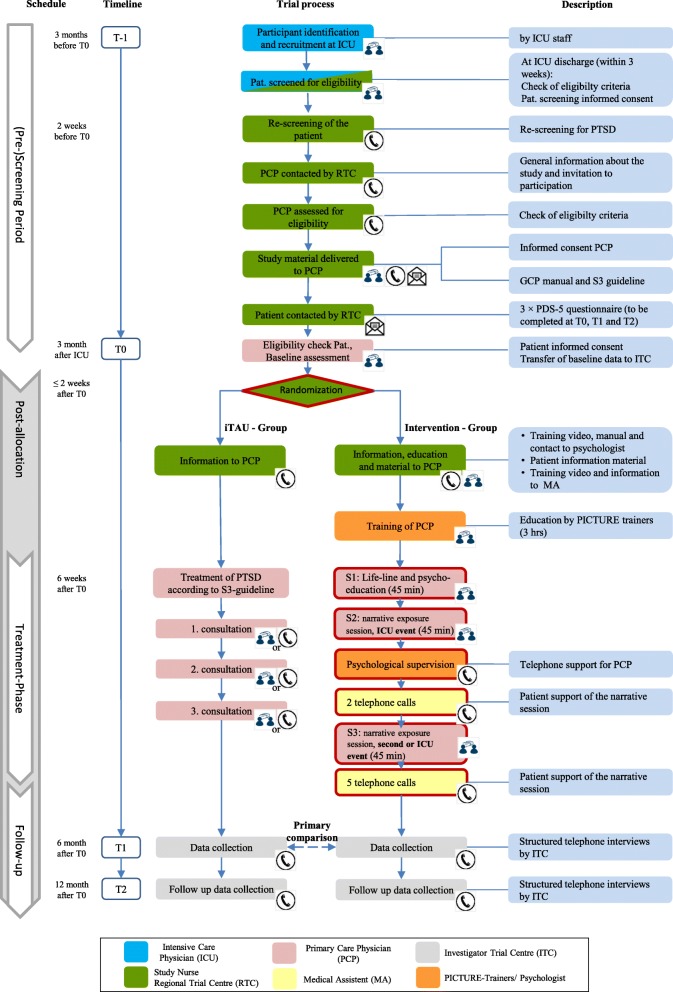
Study flow chart of the PICTURE trial: graphical depiction of study activities and components of intervention for both arms. BL baseline, GCP good clinical practice, GP general practitioner, ICU intensive care unit, ITC investigator trial center, MA medical assistant, Pat. participant, PDS Posttraumatic Stress Diagnostic Scale, PTSD posttraumatic stress disorder

**Fig. 2:**
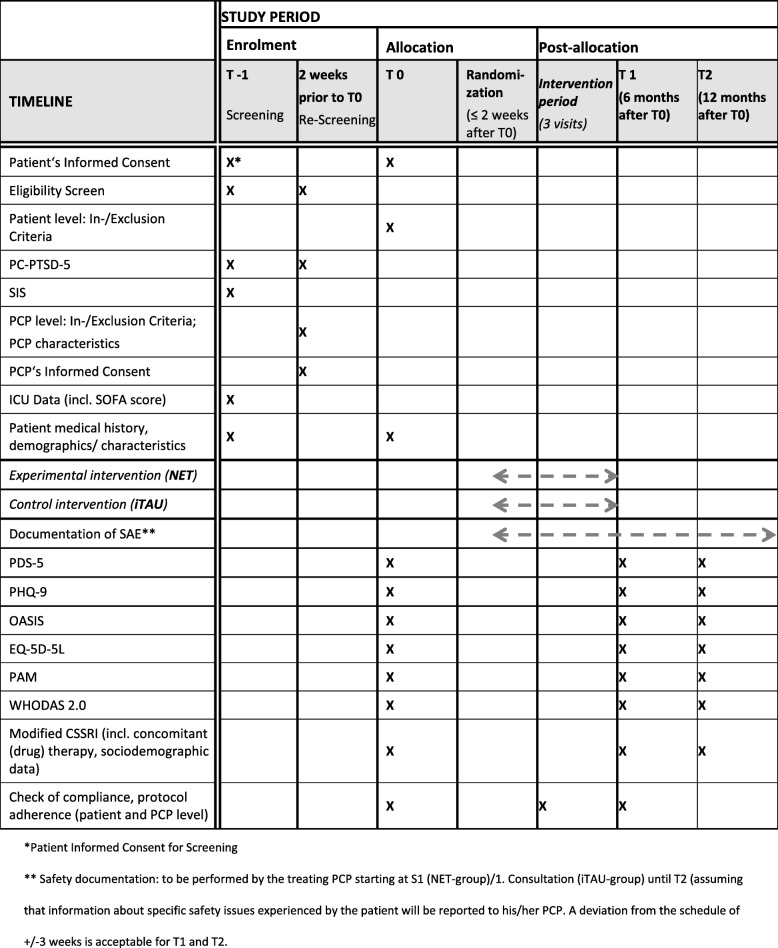
Standard protocol items (SPIRIT) for the PICTURE trial: schedule of enrolment, intervention, and assessments with their pre-planned time points T1 to T2. CSSRI Client Sociographic and Service Inventory, EQ-5D-5L Five-dimension Five-level EuroQol, ICU intensive care unit, iTAU improved treatment as usual, NET Narrative exposure therapy, OASIS Overall Anxiety Severity and Impairment Scale, PAM Patient Activation Measure, PC-PTSD Primary Care Posttraumatic Stress Disorder Screen, PCP primary care physician, PDS Posttraumatic Stress Diagnostic Scale, PHQ Patient health questionnaire, SAE serious adverse event, SIS Six-item Screener, SOFA Sequential Organ Failure Assessment, WHODAS World Health Organization Assessment Schedule

Figure 1 is a flow chart for the study. This protocol follows the “Guidance of Standard Protocol Items: Recommendations for Interventional Trials (SPIRIT) 2013 statement” [20], and it includes the schedule of enrolment and relevant assessments (Fig. 2) based on the SPIRIT figure template. A completed SPIRIT checklist is provided in Additional file 1.

### Pre-selection of eligible patients for trial recruitment and informed consent procedures

Patients will be screened by ICU staff at the time of discharge from the ICU if they meet the following criteria:

- male or female adults aged 18 to 85 years
- duration of mechanical ventilation ≥3 days
- Sequential Organ Failure Assessment (SOFA) score ≥ 5 (i.e., the maximum SOFA score during the ICU stay)
- life expectancy ≥9 months (as assessed by the intensive care physician)

Screening at ICU discharge will use a short validated paper-based questionnaire for cognition (Six-item Screener, SIS) [21] and a short validated five-item version of the Primary Care PTSD Screen (Primary Care PTSD Screen for DSM-5, PC-PTSD-5) [22].

Screened patients with a PC-PTSD-5 total score ≥3 points and no signs of significant cognitive impairment, i.e., with a SIS score ≥4 points, at ICU discharge will be re-screened 10 weeks later by the study nurse (affiliated at the corresponding trial site) via phone using the PC-PTSD-5 questionnaire only.

If a PC-PTSD-5 score of ≥3 is measured during the re-screening 10 weeks after ICU discharge, the patient will asked to attend for a baseline assessment, including confirmation of the PTSD diagnosis, at their PCP’s office. As a prerequisite for the baseline visit, the patient’s PCP will be assessed for eligibility by the study nurses of the corresponding trial site and asked for written informed consent to participate in the trial, if all inclusion criteria are met.

### Target population and eligibility criteria

#### Inclusion and exclusion criteria for patients

For final inclusion, screened patients must meet all the following inclusion criteria to be eligible for enrollment into the trial at baseline:

- PTSD symptom level: 20-item Posttraumatic Stress Diagnostic Scale for DSM-5, PDS-5 score ≥ 20 points [23]
- able to follow study instructions and likely to attend and complete all required visits and telephone surveys
- provide written informed consent

Patients are excluded from enrolment into the study if any of the following exclusion criteria apply:

- insufficient understanding of the German language
- presence of a physical or psychiatric condition that at their physician’s discretion may put the subject at risk, may confound the trial results, or may interfere with the patient’s participation in this clinical trial
- known or persistent abuse of medication, drugs, or alcohol
- major depression (PHQ-9 ≥ 23)
- acute suicidality
- life expectancy < 9 months (as assessed by the PCP)
- concomitant therapy: trauma-specific psychotherapy at baseline
- intake of any neuroleptic, anticholinergic, or anti-epileptic drugs up to 2 weeks prior to baseline
- severe PTSD symptoms (PDS-5 > 50)

#### Inclusion and exclusion criteria for PCPs

Inclusion criteria for participating PCPs are:

(1) The physician must have been registered for at least 2 years in the German statutory health-care system as a primary care physician.

(2a) The physician must have a qualification in basic psychosomatic care (Psychosomatische Grundversorgung, Bundesärztekammer, 2001) [24] to ensure that they can provide a basic level of mental health care and to ensure patient safety.

(2b) Alternatively, the PCP must have been a family doctor within the German statutory health-care system for at least 5 years with evidence of adequate psychiatric education, e.g., additional training (this is to ensure that all participating PCPs have a minimum level of psychiatric knowledge).

(3) They have provided written informed consent.

PCPs with a specialization such that more than 80% of the patients registered with their practice have a specific mental condition are excluded from the trial to ensure that enrolled practices are representative of German primary care.

### Randomization and blinding

All fully screened patients who give written informed consent for participation and who fulfill the eligibility criteria will be randomized together with the attending PCP. A full screening also includes confirmation of the PTSD diagnosis by the treating PCP of the trial participant, together with the baseline assessment at T0.

Randomization is requested by the personnel of the corresponding study site no later than 2 weeks after the baseline visit at T0. Concealed randomization to NET or iTAU will be performed with a 1:1 allocation ratio. The computer-generated randomization allocation sequence considers stratification by study site defined by the corresponding ICU. The sequence will be generated, and randomization will be performed by an independent person affiliated to the Institute for Medical Informatics, Biometry and Epidemiology of the Ludwig Maximilian University of Munich (LMU Munich) using the web-based randomization tool Randoulette [25]. The randomization list will not be accessible during the study.

After randomization, the relevant study site will have immediate access to the allocation group via online access to Randoulette and will inform the patient and the PCP practice about the respective allocation status (NET versus iTAU) via an official letter and ask the PCP practice to arrange the following appointments with the participating patient. PCPs in the intervention group will receive further information about the NET intervention and individual training.

PCPs and patients know the treatment they deliver or receive. However, this trial is designed to be *observer-blinded*. The trained interview staff affiliated to the site of the principal investigator (PI) at the Institute of General Practice and Family Medicine at LMU Munich will collect the patient-reported primary and secondary efficacy outcomes blind to group assignment. Follow-up data will be collected through structured telephone interviews at T1 and T2 without any access to additional patient data, case report forms (CRFs), or the study database. The trial statistician and the health economist will remain blinded to randomization codes throughout the trial until the study database has been finalized and locked.

### Intervention period

#### Experimental condition

After randomization, PCPs in the intervention group will receive training material (therapy manuals for PCPs and MAs, intervention videos, and a paper booklet), as well as face-to-face training by NET-qualified psychologists. In most cases, the training for PCPs will be on a one-to-one basis, though group training may also be arranged. Additionally, patients in the intervention group will receive written information about PTSD and trial procedures. Treatment in the intervention group consists of three NET sessions delivered by the PCP and case management delivered by the practice-based MA.

Furthermore, PCPs will receive training through written materials with information regarding study procedures as well as diagnostic examinations for and treatment of PTSD according to the German S3 Guideline on PTSD [10].

Three NET sessions of approximately 45 min each will be delivered by the PCP. The first session includes psychoeducation on PTSD and an overview of the patient’s biography. In this session, the patient learns about the symptoms and the theoretical background of PTSD as well as the treatment procedure. In addition, they will identify traumatic events in their biography by constructing a lifeline. In this procedure, the patient places stickers of flowers and stones, symbols of important positive and stressful events, in chronological order on a line that they construct and draw together with the PCP on a piece of paper. The line serves as a timeline, providing an overview of the patient’s biographical burden and resources. At the end of the session, the recent ICU event will be implemented in the lifeline.

In the second session, the patient will be exposed to the traumatic events in a safe environment by giving a detailed narration of their stressful experience during their ICU stay. After the second session, a qualified psychologist will provide telephone support to the PCP to review the treatment so far, prepare the third session, and give guidance and advice on the content and mode of delivery of the therapy, if necessary. Narration on another stressful key life event identified on the lifeline is recommended as the topic for the third session. Alternatively, an additional narration of the ICU event might be chosen, if no other stressful key event can be identified, or if the ICU event is still the major key event in the patient’s life.

The MAs receive written training material and personal training on case management, which is carried out by study nurses from the relevant regional trial centers. Case management consists of seven short telephone calls (approximately 15 min each), in which the MA asks about the patient’s well-being, completes a PTSD-monitoring checklist, and provides social acknowledgement of the experiences the patient had during their critical illness. MAs follow written instructions for structuring the dialog and gathering information. Included in the instructions is a color-coded rating system for the PTSD screening questionnaire results. Critical responses should prompt the MA to inform the attending PCP immediately. Questionnaire responses are noted in the booklet. Two telephone calls are made between the second and third NET sessions, followed by an additional five telephone calls between the last NET session and T1 (Fig. 1).

#### Control condition

Patients allocated to the control group will receive iTAU during at least three consultations with their PCP. Treatment will be based on current German recommendations for the diagnosis and treatment of PTSD [10] without further specifications from the study protocol. PCPs will receive written training material and detailed medical information about PTSD, based on the current national guideline for PTSD and on the application of good clinical practice in the conduct of clinical trials [26]. Because of this explicit training, we consider this treatment approach improved in comparison to treatment as usual without any additional training.

### Informed consent procedures

Prior to enrolment, straight after the eligibility of a patient has been checked and confirmed during re-screening 2 weeks before the baseline assessment at T0, the eligibility of the relevant PCP will be assessed during a telephone interview by the investigator at the regional trial center. Once the PCP’s eligibility is confirmed and they show interest in participation in the trial, both the patient and attending PCP will be provided with a full explanation of the trial in word and in writing (patient information sheet and PCP information sheet). These include detailed information about the rationale, design, conduct, potential benefits and risks, and personal implications of the trial. After the information has been provided to the patients and PCPs, they will be given sufficient time (at least 24 h) to consider participation in the trial before they are asked to do so. It is imperative that written consent is obtained before any trial-specific procedures commence. This ensures that participants have a full understanding of the trial and that the decision to participate is made voluntarily. PCPs will have the opportunity to discuss questions and concerns with the regional investigator on the phone. They will then give their patients further information about the trial and discuss open questions and concerns with them, before asking for their patient’s informed consent. All participants may withdraw their informed consent from the trial at any time and without any negative consequences for further treatment.

### Study procedures and timing schedule

The baseline assessment (T0) takes place in a PCP practice 3 months after the patient’s discharge from ICU by self-reported paper-based and interviewer questionnaires during a consultation with the PCP. In the intervention group, NET sessions start 4 weeks thereafter over a period of 6 weeks. In the control group, patients receive iTAU during three study-related consultations with their PCP within 6 months up to T1. the primary efficacy outcome will be assessed by self-reported paper-based questionnaires 6 months (T1) after the baseline visit (T0) while secondary efficacy outcomes will be assessed 6 months (T1) and 12 months (T2) after the baseline visit (T0) by observer-blinded telephone interviews. For a detailed description of study activities and the components of the intervention, see Fig. 1. The end of the clinical trial is defined by the last individual trial-specific examination during the last visit of the last patient who is participating in the trial.

### Participation discontinuation

If a patient withdraws their written informed consent or there is serious adverse event (SAE), the assigned study intervention will be discontinued for them. SAEs are defined as a patient’s death, life-threatening event, clinically relevant severe deterioration of depression or PTSD symptoms, acute suicidality, or adverse events that would constitute an unacceptable risk for the patient. The PCP will decide which incidents have to be followed up as SAEs and will report them accordingly to the PI. All SAEs will be evaluated by the PI. Additionally, a second evaluation of seriousness, causality, and expectedness will be performed by the Data and Safety Monitoring Board (DSMB) at the PI’s discretion to ensure safety evaluations follow the four-eye principle.

Whenever a patient is withdrawn from the trial, the reasons for the withdrawal or treatment discontinuation together with the corresponding dates should be recorded in detail in the patient’s medical records and the CRF. If a patient completely drops out from the study, a final examination should be conducted (e.g., by phone). In particular, every effort should be taken to assess the primary outcome. If a patient does not return for a scheduled (telephone) visit, every effort should be made to contact them to regain them for further visits according to the protocol.

For a drop-out or withdrawal of a PCP or for a SAE, immediate support will be available through the Departments of Psychiatry affiliated to the relevant regional trial center to ensure there is adequate care for patients with PTSD. The emergency back-up centers (Prof. Falkai, Department of Psychiatry and Psychotherapy, LMU Munich; Prof. Förstl, Department of Psychiatry and Psychotherapy, Technical University of Munich; Prof. Heinz, Department of Psychiatry and Psychotherapy, Universitätsmedizin Berlin, Campus Charité Mitte; Prof. Gallinat, Department of Psychiatry and Psychotherapy, University Medical Center Hamburg-Eppendorf; Prof. Bauer, Department of Psychiatry and Psychotherapy, University Hospital Carl Gustav Carus, Technische Universität Dresden; Prof. Elbert, Clinical Psychology, University of Konstanz) will be instructed accordingly. Affected trial participants will be part of the full analysis set according to the intention-to-treat (ITT) principle.

### Patient-reported outcomes

#### Primary efficacy outcome and endpoint

To evaluate the PTSD symptom severity of trial participants, the German version of the self-administered PDS-5 questionnaire will be applied. Each of the 20 items refers to PTSD-related symptoms experienced in the past month and is answered on a five-point Likert scale (from 0 not at all to 4 more than five times per week/severe) [23, 27].

The primary efficacy outcome is the PDS-5 total severity score ranging from 0 to 80 points. The pre-specified primary efficacy endpoint is the absolute change from baseline to the 6-month follow-up telephone assessment.

#### Secondary efficacy outcomes

Patient questionnaires to derive secondary efficacy outcomes were chosen based on the conceptual framework of core outcome sets [28, 29].

As depression and anxiety are known common comorbidities in patients with PTSD, trial participants are instructed to complete the primary care validated Patient Health Questionnaire 9 (PHQ-9), where each of the nine items is scored from 0 (not at all) to 3 (nearly every day), resulting in a total score of from 0 to 27 points (large scores indicate severe impairment) [30]. Anxiety will be measured by the brief Overall Anxiety Severity and Impairment Scale (OASIS) questionnaire, which contains five response options for each of the five items, coded from 0 to 4. The total score ranges from 0 (no anxiety) to 20 points, with a high score indicating severe impairment [31]. Disability will be assessed by the 12-item version of the World Health Organization Disability Assessment Schedule 2.0 (WHODAS 2.0), with a total score ranging from 0 to 100 and higher scores indicating higher levels of disability [32]. Patient activation will be measured by the 13-item Patient Activation Measure (PAM) [33], where each item scores from 1 to 4 (1 strongly disagree, 2 disagree, 3 agree, and 4 strongly agree; for the fifth item only, 5 is for not applicable). The evaluation for the latter is made by adding the raw values, which have a range of 13–52, and normalizing them to a scale of 0–100. We will use the version of the EuroQol questionnaire with five dimensions and five levels (EQ-5D-5L), which consists of the EQ descriptive system and the visual analogue scale (EQ-VAS) to measure health-related quality of life. The EQ-VAS is a thermometer-like rating scale ranging from 0 (worst imaginable health state) to 100 (best imaginable health state today) [34, 35]. Concomitant drug and non-drug therapies, and health service use will be assessed by means of a modified (shortened) German version of the Client Sociographic and Service receipt Inventory (CSSRI) [36].

For all these scores derived from the questionnaires mentioned above, the treatment effect will be assessed by means of the absolute change from baseline at months 6 and 12 for secondary efficacy outcomes. The detailed schedule of enrolment, interventions, and assessments with their pre-planned time points is shown in Fig. 2.

### Accompanying studies

#### Health economic evaluation

The objective of the health economic evaluation alongside the main trial is to assess the cost-effectiveness of the NET-oriented intervention in comparison to iTAU from a societal perspective [37]. We will consider health-care costs as well as productivity losses to describe the monetary consequences of the intervention and calculate quality-adjusted life years (QALYs) as a measure of the effects. These results will inform decision makers in the health-care sector of the economic aspects of the NET intervention and support them in deciding whether the intervention should be implemented in the German health-care system.

#### Genetic evaluation

As the first side project of the trial, we also plan to investigate the genetic distinctiveness of patients after intensive care medicine. We will consider differences in the genetic characteristics of ICU patients with PTSD compared to those of ICU patients without PTSD (not participants of the PICTURE trial). The genetic evaluation will be carried out in collaboration with the Institute of Psychiatric Phenomics and Genomics, University Hospital Munich. This project has a separate trial protocol, which has the approval of the ethics committee, and it requires separate written informed consent.

#### Process evaluation

A second side project will explore the experience of the major actors (PCPs and MAs) and patients recruited at ICUs in Berlin, Hamburg, and Dresden with the experimental intervention in the NET group. This project aims to analyze the applicability of the experimental intervention. That is, it will investigate the beneficial and obstructive factors in the effectiveness, acceptance, and feasibility of the intervention, based on the theoretical framework of acceptability [38]. Qualitative interviews will be carried out with PCPs, MAs, and patients after assessing the primary endpoint at T1 for the last patient randomized.

### Statistical planning and analyses

#### Power considerations and sample size calculation

The current literature does not provide a minimal clinically important difference for the primary outcome (PDS-5 total score for DSM-5), on which we could base the sample size calculation [39]. Therefore, we will use a calibration argument to provide a rather pragmatic minimal clinically important difference for this trial. Previous NET studies defined a decline of about 25% in the baseline score as a clinically relevant change [16]. Based on the range of the PDS-5 score from 0 to 80 points, we define 40 as the mean baseline score. Thus, a 25% change from baseline gives 10 points as the mean absolute decrease. To be more conservative (also assuming a slight effect of 4 points within the control group), we consider a difference in absolute change of 6 points between both groups as clinically relevant for these post-ICU patients. Using a standard deviation of 17, this translates into a Cohen’s *d* (standardized effect size) of 0.36. This effect is assumed to be conservative compared to the reported effects for NET [13, 40]. It can be translated into a probability of 0.6 that the observed decrease in the experimental group is larger than that in the control group (assuming a standard normal distribution). The probability of 0.6 is the target parameter needed to perform a sample size calculation with the Wilcoxon–Mann–Whitney rank-sum test. A sample size of 131 patients in each group, i.e. 262 patients in total, will have 80% power to detect a decrease of PDS-5 in the intervention group as described above compared to the control group using a Wilcoxon–Mann–Whitney rank-sum test with a 0.05 two-sided significance level (software used: nQuery Advisor 7.0). To incorporate the death of patients (resulting in the efficacy outcome being truncated due to death), we apply a non-parametric worst rank score analysis ([41, 42], for details see below) and decided to randomize an additional 78 (= 2 × 39) patients [an increase of about 30% (= 39 / 131) derived from a simulation study]. Thus, the sample size to be allocated to the trial is 2 × (131 + 39) = 340 patients in total.

It is anticipated that 3000 patients can be pre-screened, of which 1000 (33%) are expected to show posttraumatic stress [5]. The rate of non-participation is expected to be about 35%, which is a conservative assumption compared to our previous study (20% non-participants in [18]). Therefore, 650 (65%) are expected to be willing to participate (patients and their PCPs). Of these, 550 patients (about 85%) could be screened by their treating PCP 3 months post-ICU discharge (assuming a mortality rate after 6 months of about 15% as in [18]), 400 (about 70%) could meet the inclusion criteria, and 340 (85%) patients (and their PCPs) could consent to participate in the study at the baseline visit. We assume a 30% drop-out rate from baseline over the 6 months before the primary endpoint is assessed. There is no pre-planned interim efficacy analysis and no sample size recalculation.

#### Statistical analyses for primary and secondary endpoints

The primary efficacy endpoint is the absolute change in the PDS total severity score from baseline at month 6: ΔPDS = PDS(T1) – PDS(T0).

By default, the mode of administration is a self-administered paper-based version. For patients who do not complete and send back the paper-based patient questionnaire (non-responding survivors), the PDS-5 total score will be assessed during the telephone surveys scheduled 6 months (T1) and 12 months (T2) after randomization.

It is assumed that death is the most likely cause of missingness. Therefore, a composite endpoint approach will be applied, combining information about the change in PDS total score and mortality into a single variable [41].

The null hypothesis, *G*_NET_(*x*) = *G*_iTAU_(*x*) and *K*_NET_(*t*) = *K*_iTAU_(*t*) (0 < *t* ≤ *T*, date of death), implies that treatment groups NET and iTAU will not differ with respect to the distributions of the observed outcome measure ΔPDS. Here, *G*(*x*) is the cumulative probability distribution of the observed change in PDS severity scores at T1 in groups NET or iTAU, and the distribution *K*(*t*) of the date of death is the cumulative distribution of informative event times for the compared groups.

The null hypothesis will be tested by a non-parametric approach using a modified version of the Wilcoxon–Mann–Whitney *U* test, which basically allocates the tied worst ranks to all missing values (the worst rank score analysis was proposed by Lachin [42]). The null hypothesis can be rejected if the two-sided *p* value related to the test statistic for the treatment effect is equal to or smaller than the significance level α = 0.05. This test strategy is tailored to a particular alternative hypothesis, i.e., (i) NET will either be superior to iTAU in terms of ΔPDS, but with no impact on survival, (ii) NET will be superior to iTAU in terms of survival, but with no impact on ΔPDS, or (iii) NET will be superior to iTAU for both ΔPDS and survival.

If the PDS total severity scores are not informative for future death events, the worst rank replacement will simply lead to a power loss and no inflation in the type I error rate. Should the PDS total severity scores be informative for future death events, the worst rank replacement will result in an unbiased test of a particular alternative [42].

The principal analysis will be performed according to the ITT principle, and not adjusted for screening or baseline covariates or site. The significance level is set to alpha = 5% (two-sided).

Missing data prior to the follow-up measurement will occur because of an informative disease-related event (e.g., death or morbidity) or for other reasons (e.g., non-responders at follow-up measurements at T1 and T2, loss to follow-up, or consent withdrawn). To address the impact of several missingness mechanisms (missing at random or missing not at random), sensitivity analyses will be performed: mixed effect models assuming missing at random using the whole observed longitudinal PDS profile of the surviving patient; multiple imputations techniques; or even complete case analyses using the analysis of covariance (absolute change score as the response variable and treatment group as the covariate, adjusting for the baseline score value) for responding survivors until T1.

Moreover, sensitivity analyses will be performed in the per-protocol population using linear mixed effects models to explore the role of covariates (e.g., patient age and gender).

The full statistical analysis plan will be finalized and revised in a blinded manner ahead of the database lock after the last patient’s last 12-month telephone call.

#### Definition of analysis data sets

Each trial participant’s allocation to the different analysis populations (full analysis data set according to the ITT principle, per-protocol analysis data set, and safety analysis data set) will be defined and explained in the statistical analysis plan, which will be finalized before the analysis. During the data review, deviations from the protocol will be assessed as minor or major. Major deviations from the protocol will lead to the exclusion of a participant from the per-protocol analysis data set. The full analysis data set according to the ITT principle will consider all randomized patients with at least one study-related visit at a physician’s office during the intervention period (for the NET group, at least one NET session and for the iTAU group, at least one face-to-face consultation). Furthermore, patients who die before the evaluation of efficacy outcomes (truncation due to death) are part of the principal analysis incorporating the timing of death of the trial participant.

### Safety assessment and reporting of adverse events

Overall, a low frequency of SAEs can be expected due to the narrative exposure itself. SAEs are events that (1) result in death, (2) are life-threatening, (3) require hospitalization or cause prolongation of existing hospitalization, (4) result in persistent or significant disability or incapability, (5) are a congenital anomaly or birth defect, or (6) require intervention to prevent permanent impairment or damage. SAEs will be regularly monitored and investigated from the start of the intervention at Session 1 in the NET group and from the first of the three PCP consultations in the iTAU-group until the end of the trial at T2. The PCP will decide which events have to be followed up as SAEs and will report them accordingly to the PI.

The PCP is the first point of contact during the intervention period, since the telephone interviews are at T1 and T2. If a patient cannot be reached via telephone at T1 and T2, the RTC will contact the respective PCP for further information on the patient’s possible SAE status. For the whole trial duration from T0 up to T2, the PCP is instructed to report all SAEs, or the relocation or death of the patient proactively. Since in Germany the PCP is the first point of contact to receive updates from hospitals, specialists, or other medical services involved in the patient’s care, this should enable us to monitor patient safety continuously. In addition, psychiatric back-up clinics are available at each site for emergency cases. All SAEs will be reported to the PI and the DSMB.

Since there is a great heterogeneity in adverse events in primary care, it is sometimes not possible to differentiate between adverse events and pure signs of discomfort in patients [43]. Therefore, we decided not to assess any adverse events. All documented SAEs will be listed by study site and patient and displayed in summary tables. The incidence of SAEs and their relationship to the assigned intervention will be descriptively analyzed [44, 45].

### Data management

The Institute of General Practice and Family Medicine, University Hospital, LMU Munich, as the coordinating study center, is responsible for data management, which encompasses all tasks concerning processing and utilization of study data, with the aim of guaranteeing high-quality data and providing a valid study database for statistical analyses. All data management activities will be done according to the current standard operating procedures of the investigational trial center (ITC).

#### Data collection and transmission

All data collected during the trial will be documented using electronic case report forms (eCRFs). The source data will be stored regionally in the patients’ files. Clinical and patient-reported outcome data will be collected by the ITC in Munich at the site of the PI via self-administered questionnaires and via phone interviews at T1 and T2. ITC staff are blinded to the assigned treatment given to the interviewed patient.

### Data handling

Data collection will be managed using a secure, web-based system (OpenClinica© Community Edition, Version 3.12). Data input requires an internet connection and a browser. Authorization and the electronic signature of users is granted via a login and password. To ensure the security of the data entered, web access is encrypted via SSL certificates. All data collected throughout the study period will be stored in a secure server at the Leibniz Supercomputing Centre of the Bavarian Academy of Sciences and Humanities (Leibniz-Rechenzentrum, LRZ). A secure file folder will be constructed before initiation of the trial. Access is limited to the PI and the data manager. Study participants will be identifiable through their study-specific screening number. Data routinely collected from patients, including questionnaire data, will be stored at the trial site up to T1 and at the coordinating trial center in Munich at T1 and T2, using eCRFs.

Any changes made during data collection will be documented using audit trails in OpenClinica. Data integrity is enforced by referential data rules, valid values, range checks, and consistency checks against data already stored in the database. Plausibility checks will be applied during data entry and before the data are transferred to the database. To ensure valid comparable data, data cleaning is carried out according to a data validation plan. After the database is locked, all study data will be exported from OpenClinica© for statistical analyses using SAS (Institute Inc., Cary, NC, USA) or the software package R, version 3.5.0 or higher (www.R-project.org).

#### Monitoring

An independent clinical monitor will check for accuracy, completeness, consistency, and reliability of the eCRFs by comparing documented data with source data. The monitor will check that data are collected, stored, and managed appropriately at all trial sites. Additionally, the monitor will check SAE documentation and status as well as documentation and follow-up of protocol deviations. Monitoring visits will be carried out regularly according to the standard operating procedures at each trial site independently, to ensure the trial procedure is executed according to good clinical practice [26].

### Data safety and monitoring board

An independent DSMB has been established to monitor the course of the study, recruitment, patient safety, the integrity of the trial, and if necessary to give a recommendation to the coordinating investigator and sponsor for discontinuation, modification, or continuation of the study. Furthermore, the DSMB will periodically review the safety-relevant events reported to this board. The members of the DSMB are Dr. Jochem König (Mainz), Dr. Andreas Linde (Königsfelden), Prof. Wolfgang Miltner (Jena), and Prof. Frank Schneider (Aachen).

## Discussion

The aim of the PICTURE trial is to evaluate the effect of a multicomponent primary-care-based intervention for ICU survivors suffering from posttraumatic stress. Since PTSD after critical illness is still an underestimated problem and PCPs are the first point of contact for providing health care to these patients, it might be beneficial to investigate this disease in ICU survivors and for the PCP to acquire new non-pharmacological treatment options to help these patients quickly during the typically long waiting periods for specialist support and therapy. Therefore, it is important to assess the effects of NET adapted to the primary care setting. The patient and the attending PCP are the information units within this trial. A single PCP will treat only one ICU patient (i.e. the first to be randomized). Therefore, all conclusions from the PICTURE trial will be limited to the pair of patient and PCP.

Assuming a representative population of PTSD patients and a representative population of ICU patients, the effect may be interpretable in a generalizable way, and it may reflect a general statement about the efficacy of a German, randomly chosen PCP who meets a randomly chosen patient. This generalizability might be reduced by specific selection processes (e.g., PCPs eager to join the trial, the long-term effect of the training, whether the PCP is eager to learn more about NET, whether there is a declining efficacy curve for PCPs, or how MAs deliver the phone support, which is the second component of the experimental intervention). These also need to be elucidated in specific sensitivity and process analyses.

Furthermore, this is a complex intervention and claims cannot be linked or partitioned into specific components. However, the involvement of physicians in primary care also poses certain challenges, as doctors usually have no experience in conducting clinical trials, which might make it difficult to implement certain study procedures. For this reason, before the beginning of the intervention phase, the participating physicians will be trained not only in study-specific procedures but also in the basics of good clinical practice as prescribed by the International Council for Harmonisation of Technical Requirements for Pharmaceuticals for Human Use.

PICTURE may have further limitations. A selection bias of participating PCPs and patients may limit the generalizability of the results. The control group delivers iTAU, which might not be representative for usual care in general but might be more thorough and conscientious. Even though the applied NET is adapted to the primary care setting, there may still be barriers to implementation in daily clinical practice, e.g. due to limited time resources in PCP practices. If two or more participating PCPs from the same practice have patients assigned to different treatment groups, there may be contamination between the intervention and control participants. If a PCP has more than one relevant patient, only the first patient randomized will be included in the full analysis data set. We expect this scenario to be rather unlikely, and it would lead to individual randomization instead of a cluster randomized design.

A major risk in the execution of the study could be insufficient recruitment due to the gradual integration of patients. To reduce this risk, we intensified the screening and recruitment procedure carried out in the SMOOTH trial, which was performed in and around Jena and Berlin, by increasing the number of recruitment areas to Berlin, Dresden, Hamburg, Tübingen, and Munich. In each catchment area, we employ study nurses to monitor and support screening and recruitment. The risk of adoption (learning) of the intervention by PCPs may lead to heterogeneity in intervention delivery. We may be able to reduce heterogeneity in the intervention by limiting the number of patients for each PCP (one patient per PCP).

### Trial status

At the time of manuscript submission, the study design has been evaluated by an independent international reviewer and has been approved by the ethics committee of LMU Munich. The first patient was pre-screened at an ICU at the end of October 2017 with the opening of the trial site of the PI (start of patient recruitment) in Munich. Until 26 April 2018, no study participants have been randomized. We expect enrolment of the first patient in summer 2018.

### Protocol version

Version 3.0, 14 March 2018.

## Additional file

Additional file 1: SPIRIT 2013 Checklist. (DOC 120 kb)

## Abbreviations

(e)CRF: (Electronic) case report form
AE: Adverse event
CSSRI: Client Sociographic and Service Inventory
DSM: Diagnostic and Statistical Manual
DSMB: Data safety and monitoring board
EQ-5D-5L: Five-dimension Five-level EuroQol
EQ-VAS: EuroQol Visual Analog Scale
IBE: Institute for Medical Information Processing, Biometry and Epidemiology
ICU: Intensive care unit
iTAU: Improved treatment as usual
ITC: Investigational trial center
ITT: Intention to treat
LMU Munich: Ludwig Maximilian University of Munich
MA: Medical assistant
NET: Narrative exposure therapy
OASIS: Overall Anxiety Severity and Impairment Scale
PAM: Patient Activation Measure
PCP: Primary care physician
PC-PTSD: Primary Care PTSD Screen
PDS: Posttraumatic Stress Diagnostic Scale
PHQ: Patient Health Questionnaire
PI: Principal investigator
PTSD: Posttraumatic stress disorder
QALY: Quality-adjusted life year
S: Session
SAE: Serious adverse event
SIS: Six-item Screener
SOFA: Sequential Organ Failure Assessment
TFA: Theoretical framework of acceptability
VAS: Visual analogue scale
WHODAS: World Health Organization Assessment Schedule
